# A Tale of Two Periods: The Evolution of Determinants and CVD Mortality Risk in Metastatic NSCLC

**DOI:** 10.31083/RCM39296

**Published:** 2025-09-28

**Authors:** Junyan Xia, Yanting Pang, Yiqiu Yang, Jun Teng, Qian Lin

**Affiliations:** ^1^Department of Cardiology, Dongzhimen Hospital, Beijing University of Chinese Medicine, 100700 Beijing, China; ^2^Graduate School, Beijing University of Chinese Medicine, 100029 Beijing, China; ^3^Department of Hematology and Oncology, Dongzhimen Hospital, Beijing University of Chinese Medicine, 100700 Beijing, China; ^4^Respiratory Disease Center, Dongzhimen Hospital, Beijing University of Chinese Medicine, 100700 Beijing, China

**Keywords:** non-small cell lung cancer, SEER database, immunotherapy, cardiovascular mortality risk, accelerated failure time model, CDC-WONDER database

## Abstract

**Background:**

Immunotherapy has redefined the treatment era for metastatic non-small cell lung cancer (NSCLC); therefore, this study aimed to explore trends in survival and cardiovascular disease (CVD) mortality risk before and after the widespread adoption of immunotherapy.

**Methods:**

This research utilized information from the Surveillance, Epidemiology, and End Results (SEER) program of the National Cancer Institute and the Wide-Ranging Online Data for Epidemiologic Research (CDC-WONDER) database from the Centers for Disease Control and Prevention. The study population comprised patients with metastatic NSCLC from the pre- (2011–2014) and post-immunotherapy (2016–2019) periods. Survival determinants and CVD mortality trends were analyzed using propensity score matching, Kaplan–Meier survival analyses, competing risk models, and accelerated failure time (AFT) models.

**Results:**

A total of 78,028 metastatic NSCLC patients were enrolled in the study, with significant improvements noted in overall survival (OS) and cancer-specific survival (CSS) in the later stages of immunotherapy. The AFT model analysis identified treatment modality, pathological subtype, metastatic site, and some non-medical factors as survival determinants. The interaction analyses revealed that the survival differences among certain subgroups intensified in the post-immunotherapy period. Despite the lack of significant differences in CVD mortality and subgroup composition between the two periods, CVD mortality risk remained high compared with the general U.S. population.

**Conclusion:**

Survival of patients with metastatic NSCLC has improved significantly since the introduction of immunotherapy. However, survival differences between some subpopulations continue to intensify, while CVD mortality risk also remains a key concern.

## 1. Introduction

Lung cancer remains the leading cause of cancer-related mortality in the United States, accounting for an estimated 125,070 deaths in 2024 alone, with non-small cell lung cancer (NSCLC) comprising approximately 85% of all cases [1, 2]. Despite advancements in early screening and diagnostic strategies, the incidence of advanced and metastatic NSCLC remains substantial [3, 4]. A major turning point in NSCLC treatment occurred with the approval and integration of immune checkpoint inhibitors (ICIs) into clinical practice around 2015, which has since transformed the therapeutic landscape and extended survival in select patient populations [5]. However, the degree of benefit from these therapeutic advances appears to vary across subgroups, underscoring the importance of understanding survival determinants in the evolving treatment era.

Survival outcomes in metastatic NSCLC are influenced by a range of clinical and non-clinical factors. Clinical variables such as age, sex, tumor histology, metastatic sites, and treatment modalities (e.g., chemotherapy, radiotherapy) are well-documented contributors to prognosis [6]. Equally important, yet less frequently studied in temporal contexts, are non-medical factors including race, socioeconomic status, marital status, and geographic location [7, 8]. These determinants may interact with medical care access, treatment adherence, and psychosocial support, contributing to outcome disparities. Given the dynamic nature of both therapeutic innovation and health equity over time, investigating how these determinants evolve across distinct treatment eras is crucial for optimizing care strategies in metastatic NSCLC.

In addition to survival disparities, emerging treatment-related toxicities—particularly cardiovascular disease (CVD)—have raised new concerns in the immunotherapy era [9, 10]. CVD in this population may stem from both shared risk profiles (e.g., aging, smoking) and therapy-induced cardiotoxicity [11, 12]. Although some studies have assessed CVD mortality risk across treatment modalities, their scope has often been limited by short follow-up periods and narrow population subsets [13, 14, 15]. Therefore, leveraging real-world data to evaluate trends in both survival determinants and CVD mortality before and after the adoption of immunotherapy can offer valuable insights for patient stratification and long-term care planning. The present study aims to (1) examine the temporal evolution of survival determinants in metastatic NSCLC and (2) update the evidence on CVD mortality risk using data from the Surveillance, Epidemiology, and End Results (SEER) Program and the Centers for Disease Control and Prevention Wide-Ranging Online Data for Epidemiologic Research (CDC-WONDER) databases.

## 2. Methods

### 2.1 Data Source

This study employed the November 2022 Submission dataset from the SEER Program, US National Cancer Institute, to aggregate population-based cancer data across 17 registries, covering about 26.5% of the US population. Heart disease mortality data for the US populace were derived from the CDC-WONDER database (underlying cause of death, 1999–2020). The Ethics Committee of Dongzhimen Hospital, Beijing University of Chinese Medicine granted the ethical exemption, citing the anonymization and public availability of the data from both databases.

### 2.2 Study Population

Employing the International Classification of Diseases for Oncology, Third Edition (ICD-O-3) as delineated in **Supplementary Table 1**, this research identified individuals with a single primary NSCLC in the SEER database across pre- (2011–2014) and post-immunotherapy (2016–2019) periods, totaling 204,038 patients. Cohorts were categorized by immunotherapy period, adhering to consistent exclusion criteria: Tumor, Node, Metastasis (TNM) stages I–III or unspecified, surgical treatment, unrecorded or zero survival months, and ages outside 18–84 (**Supplementary Fig. 1**). For comparative analysis, CVD mortality data for the U.S. population aged 18–84 were extracted from the CDC-WONDER database for 2011–2014 and 2016–2019, respectively, to match the pre- and post-immunotherapy periods in the SEER NSCLC cohort and minimize year- and population-related bias.

### 2.3 Key Variables

The extracted demographic and clinical parameters encompassed age (18–84 years), sex (female, male), race (white, black, other), laterality (left, right, other), pathology subtype (lung adenocarcinoma (LUAD), lung squamous cell carcinoma (LUSC), other), metastasis site (bone, brain, liver, lung), therapeutic approaches (radiotherapy, chemotherapy), marital status (married, other), median household income (<USD 50,000, USD 50,000–USD 75,000, >USD 75,000), and geographic location of residence (metropolitan, non-metropolitan).

Our investigation’s primary outcomes of interest were overall survival (OS) and cancer-specific survival (CSS), alongside assessing cardiovascular risk across pre- and post-immunotherapy periods through the lens of CVD mortality. OS refers to the time from diagnosis to any-cause death, CSS to cancer-caused death, and Survival Time to the period from diagnosis to death or last follow-up (December 31, 2020). CVD mortality was determined based on the underlying cause of death recorded in death certificates, as documented in the SEER and CDC-WONDER databases and classified according to ICD-10 codes, including diseases of the heart (I01–I02, I05–I09, I20–I28, I30–I52), hypertension without heart disease (I10–I15), cerebrovascular diseases (I60–I69), other diseases of arteries, arterioles, and capillaries (I70–I78), and other unspecified disorders of the circulatory system (I95–I99) [16].

### 2.4 Statistical Analysis

Continuous variables were described using mean ± standard deviation and analyzed with an equal-variance *t*-test, while categorical variables were presented as percentages and assessed through the chi-square test. To align cohorts’ pre- and post-immunotherapy periods on a 1:1 basis, a propensity score matching (PSM) method with the nearest neighbor approach was utilized, setting a caliper of 0.1 and avoiding replacement techniques [17]. Survival curves were generated pre-and post-PSM adjustment utilizing the Kaplan-Meier estimator, with the log-rank test evaluating differences in OS and CVD mortality between groups. Fine-gray-based competitive risk analysis were then used to assess cumulative mortality from CSS and CVD [18].

Given that several covariates violated the proportional hazards assumption (as assessed by Schoenfeld residuals and visual inspection), we adopted an accelerated failure time (AFT) model to evaluate survival duration. Among tested distributions (lognormal, Weibull, exponential), the lognormal AFT model provided the best fit, based on the lowest Akaike Information Criterion (AIC) and Bayesian Information Criterion (BIC) values (**Supplementary Fig. 2, Supplementary Table 2**). This model facilitates interpretation beyond the constraints of the proportional hazards assumption. In this context, the exponentiated coefficients are expressed as time ratios (TRs), which quantify how a given covariate alters the predicted survival duration. Specifically, a TR >1 suggests that the covariate is associated with prolonged survival time (i.e., the event is expected to occur later), whereas a TR <1 indicates an association with shorter survival time (i.e., earlier event occurrence) [19, 20]. Covariates with a univariate *p* < 0.1 were included in the multivariate AFT analysis to identify independent prognostic factors and potential effect modifications across the pre- and post-immunotherapy periods.

CVD mortality age distribution disparities necessitated the computation of a weighted mean for CVD mortality, adjusting for age distribution weights to furnish an accurate CVD death risk comparison in NSCLC patients versus the general U.S. population. R (version 4.5.1; R Core Team, R Foundation for Statistical Computing, Vienna, Austria) was used for all statistical computations, employing two-sided tests and considering *p* < 0.05 as the threshold for statistical significance.

## 3. Results

### 3.1 Cohort Characteristics

A total of 78,028 patients with single primary stage IV NSCLC were included, with an average age of (66.1 ± 10.2) years, of which 45.6% were female. The patients were divided into two cohorts: 40,135 in the pre-immunotherapy period and 37,893 in the post-immunotherapy period (Table 1). Significant differences were noted between the cohorts in various factors such as age, sex, race, laterality, pathology subtype, therapeutic approaches (radiotherapy, chemotherapy), metastasis site (bone, brain), and median household income (all *p* < 0.001). After performing PSM at a 1:1 ratio, each cohort was adjusted to have 33,481 patients (Table 1). Subsequent analysis post-PSM showed standardized mean differences (SMDs) for each variable below 0.1, indicating a high level of similarity in the distributions of the two cohorts (**Supplementary Fig. 3**).

**Table 1.** **Baseline characteristics of metastatic NSCLC patients before and after propensity score matching (PSM)**.

Variables		Original Data					PSM Data				
		Pre	Post	Total	Test Statistic	*p* value	Pre	Post	Test Statistic	*p* value	SMD
		n = 40,135	n = 37,893	n = 78,028	(t, χ^2^)^*^		n = 33,481	n = 33,481	(t, χ^2^)		
Age (mean (SD))		65.8 (10.3)	66.5 (10.0)	66.1 (10.2)	–10.366	<0.001	66.0 (10.3)	66.2 (10.0)	–2.493	0.013	0.019
Sex (%)					13.217	<0.001			0.001	0.981	<0.001
	Female	18,034 (44.9)	17,519 (46.2)	35,553 (45.6)			15,251 (45.6)	15,255 (45.6)			
	Male	22,101 (55.1)	20,374 (53.8)	42,475 (54.4)			18,230 (54.4)	18,226 (54.4)			
Race (%)					137.290	<0.001			1.996	0.369	0.011
	White	30,917 (77.0)	28,193 (74.4)	59,110 (75.8)			25,312 (75.6)	25,408 (75.9)			
	Black	5206 (13.0)	4916 (13.0)	10,122 (13.0)			4469 (13.3)	4346 (13.0)			
	Others	4012 (10.0)	4784 (12.6)	8796 (11.3)			3700 (11.1)	3727 (11.1)			
Laterality (%)					16.562	<0.001			4.468	0.107	0.016
	Left	15,388 (38.3)	14,671 (38.7)	30,059 (38.5)			12,965 (38.7)	12,948 (38.7)			
	Right	21,788 (54.3)	20,710 (54.7)	42,498 (54.5)			18,063 (54.0)	18,215 (54.4)			
	Others	2959 (7.4)	2512 (6.6)	5471 (7.0)			2453 (7.3)	2318 (6.9)			
Hist (%)					456.779	<0.001			39.995	<0.001	0.049
	LUAD	24,324 (60.6)	25,321 (66.8)	49,645 (63.6)			20,943 (62.6)	21,612 (64.6)			
	LUSC	7628 (19.0)	6931 (18.3)	14,559 (18.7)			6401 (19.1)	6313 (18.9)			
	Others	8183 (20.4)	5641 (14.9)	13,824 (17.7)			6137 (18.3)	5556 (16.6)			
Radiation (%)					25.063	<0.001			0.850	0.357	0.007
	No/Unknown	20,912 (52.1)	20,423 (53.9)	41,335 (53.0)			17,809 (53.2)	17,929 (53.5)			
	Yes	19,223 (47.9)	17,470 (46.1)	36,693 (47.0)			15,672 (46.8)	15,552 (46.5)			
Chemotherapy (%)					117.901	<0.001			3.390	0.066	0.014
	No/Unknown	15,541 (38.7)	16,121 (42.5)	31,662 (40.6)			13,491 (40.3)	13,726 (41.0)			
	Yes	24,594 (61.3)	21,772 (57.5)	46,366 (59.4)			19,990 (59.7)	19,755 (59.0)			
DX.bone (%)					116.474	<0.001			2.277	0.131	0.012
	No/Unknown	24,424 (60.9)	21,618 (57.1)	46,042 (59.0)			19,878 (59.4)	19,685 (58.8)			
	Yes	15,711 (39.1)	16,275 (42.9)	31,986 (41.0)			13,603 (40.6)	13,796 (41.2)			
DX.brain (%)					61.455	<0.001			1.331	0.249	0.009
	No/Unknown	28,959 (72.2)	26,374 (69.6)	55,333 (70.9)			23,951 (71.5)	23,815 (71.1)			
	Yes	11,176 (27.8)	11,519 (30.4)	22,695 (29.1)			9530 (28.5)	9666 (28.9)			
DX.liver (%)					2.326	0.127			2.841	0.092	0.013
	No/Unknown	33,098 (82.5)	31,090 (82.0)	64,188 (82.3)			27,423 (81.9)	27,591 (82.4)			
	Yes	7037 (17.5)	6803 (18.0)	13,840 (17.7)			6058 (18.1)	5890 (17.6)			
DX.lung (%)					0.825	0.364			1.272	0.259	0.009
	No/Unknown	28,101 (70.0)	26,645 (70.3)	54,746 (70.2)			23,302 (69.6)	23,437 (70.0)			
	Yes	12,034 (30.0)	11,248 (29.7)	23,282 (29.8)			10,179 (30.4)	10,044 (30.0)			
MaritalStatus (%)					0.000	0.993			0.174	0.676	0.003
	Married	20,601 (51.3)	19,448 (51.3)	40,049 (51.3)			17,207 (51.4)	17,152 (51.2)			
	Others	19,534 (48.7)	18,445 (48.7)	37,979 (48.7)			16,274 (48.6)	16,329 (48.8)			
Income (%)					1677.066	<0.001			38.447	<0.001	0.048
	USD 50,000-	6919 (17.2)	4831 (12.7)	11,750 (15.1)			5298 (15.8)	4820 (14.4)			
	USD 50,000–USD 75,000	21,620 (53.9)	16,830 (44.4)	38,450 (49.3)			16,720 (49.9)	16,596 (49.6)			
	USD 75,000+	11,596 (28.9)	16,232 (42.8)	27,828 (35.7)			11,463 (34.2)	12,065 (36.0)			
County (%)					1.079	0.299			0.761	0.383	0.007
	Metropolitan	34,076 (84.9)	32,274 (85.2)	11,678 (15.0)			28,286 (84.5)	28,203 (84.2)			
	Nonmetropolitan	6059 (15.1)	5619 (14.8)	66,350 (85.0)			5195 (15.5)	5278 (15.8)			

^*^t test for continuous variables, Pearson’s χ^2^ test for categorical variables; Abbreviations: NSCLC, non-small cell lung cancer; LUAD, lung adenocarcinoma; LUSC, lung squamous cell carcinoma; PSM, propensity score matching; SD, standard deviation; SMD, standardized mean difference; Hist, histology; DX, diagnosis of metastasis; Pre, pre-immunotherapy period; Post, post-immunotherapy period.

### 3.2 Survival Outcomes

**Fig. 1.**
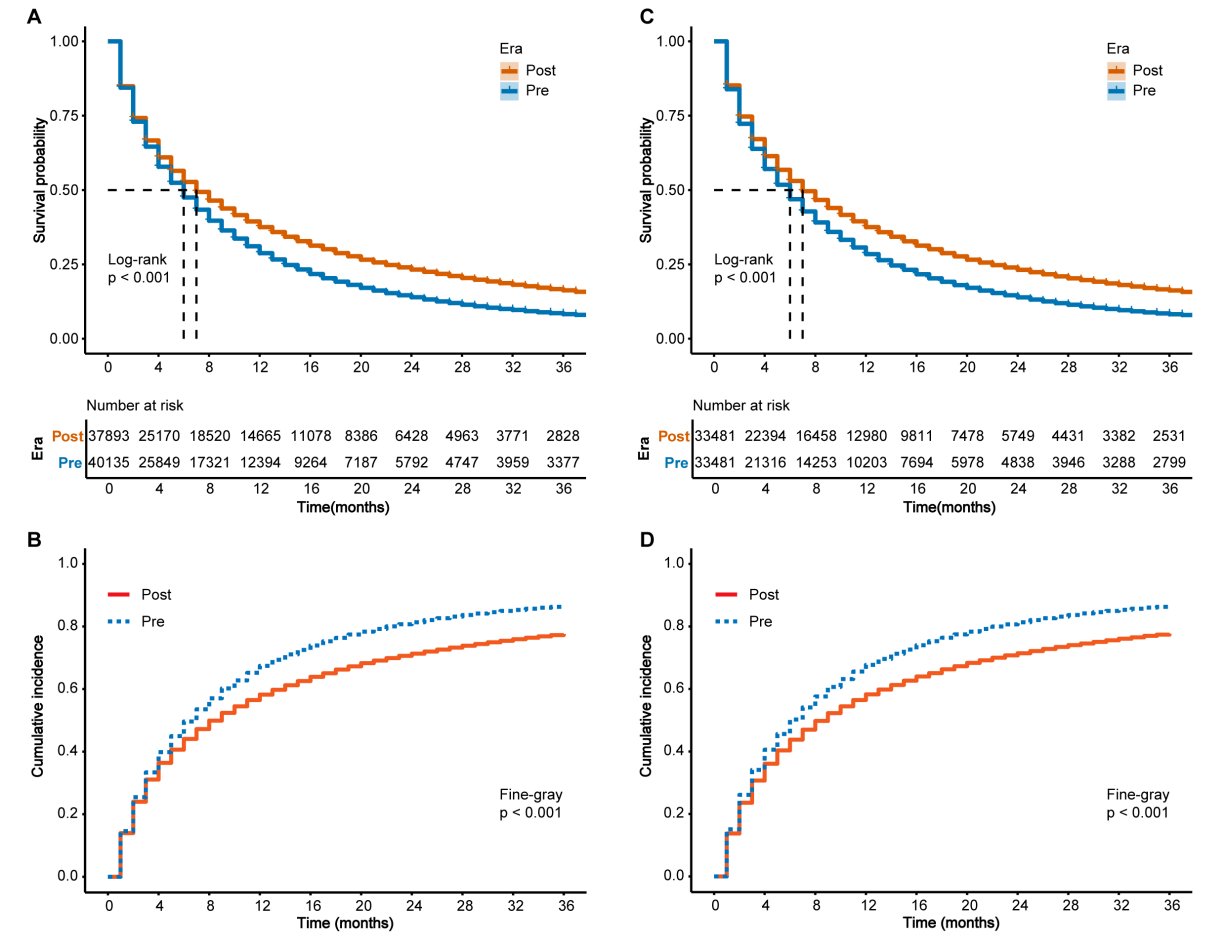
**Kaplan–Meier survival curves and competing risk models in patients with metastatic non-small cell lung cancer (NSCLC), before and after propensity score matching (PSM)**. (A) Kaplan–Meier curves for overall survival (OS) before PSM. (B) Competing risk model for cancer-specific survival (CSS) before PSM. (C) Kaplan–Meier curves for OS after PSM. (D) Competing risk model for CSS after PSM. Log-rank and Fine–Gray tests were used to assess statistical significance. Pre, pre-immunotherapy period; Post, post-immunotherapy period.

Before PSM, Kaplan-Meier survival analysis revealed that patients in the pre-immunotherapy period experienced significantly declined OS compared to those in the post-immunotherapy period (*p* < 0.001, Fig. 1A). Competitive risk analysis further demonstrated a notable increase in cumulative CSS mortality in the pre-immunotherapy period relative to the post-immunotherapy period (*p* < 0.001, Fig. 1B). Following PSM, this advantage persisted, with significant differences in OS and CSS mortality still evident (all *p* < 0.001, Fig. 1C,D). Post-PSM subgroup analysis showed that the median OS and CSS of LUAD and LUSC patients were lower in the pre-immunotherapy period than in the post-immunotherapy period (all *p* < 0.001) (**Supplementary Table 3**).

### 3.3 AFT Analysis

Univariate and multivariate AFT analyses on post-PSM data for OS and CSS (Tables 2,3) revealed significant factors affecting survival: age, period, sex, race, laterality, pathology subtype, treatment approaches (radiotherapy, chemotherapy), metastasis site (bone, brain, liver), marital status, median household income, and geographic location of residence (*p* < 0.05). Factors with *p* < 0.1 in univariate analysis were included in multivariate analysis, confirming their independent impact on OS and CSS. Positive influences on prolonged survival included the post-immunotherapy period, non-white race, chemotherapy, radiotherapy, higher median household income (>USD 50,000), and metropolitan residency. Negative influences encompassed advanced age, male sex, right-sided and unspecified tumor locations, bone, brain, and liver metastases, and non-married status. The post-immunotherapy period significantly improved OS (26.9% increase, TR = 1.269, 95% CI 1.247–1.292) and CSS (29.0% increase, TR = 1.290, 95% CI 1.267–1.314) compared to the pre-immunotherapy period.

**Table 2.** **Accelerated failure time (AFT) model for overall survival (OS) in patients with metastatic NSCLC after PSM**.

Variables		Univariable						Multivariable					
		Estimate	Std.Err.	Z value	TR	95% CI	*p* value	Estimate	Std.Err.	Z value	TR	95% CI	*p* value
Age		–0.019	0.001	–38.163	0.981	0.980, 0.982	<0.001	–0.012	0.000	–24.879	0.988	0.988, 0.989	<0.001
Era													
	Post	0.240	0.010	22.938	1.271	1.245, 1.297	<0.001	0.238	0.009	26.281	1.269	1.247, 1.292	<0.001
Sex													
	Male	–0.267	0.010	–25.536	0.766	0.750, 0.782	<0.001	–0.239	0.009	–25.730	0.788	0.773, 0.802	<0.001
Race													
	Black	–0.044	0.015	–2.829	0.957	0.929, 0.987	0.005	0.002	0.014	0.154	1.002	0.975, 1.030	0.877
	Others	0.487	0.017	28.840	1.628	1.575, 1.683	<0.001	0.337	0.015	22.315	1.401	1.360, 1.443	<0.001
Laterality													
	Right	–0.024	0.011	–2.220	0.976	0.955, 0.997	0.026	–0.037	0.010	–3.836	0.964	0.946, 0.982	<0.001
	Others	–0.150	0.021	–7.041	0.861	0.826, 0.898	<0.001	–0.073	0.019	–3.959	0.929	0.896, 0.964	<0.001
Hist													
	LUSC	–0.357	0.014	–26.427	0.700	0.682, 0.719	<0.001	–0.222	0.012	–18.209	0.801	0.782, 0.821	<0.001
	Others	–0.354	0.014	–25.314	0.702	0.683, 0.722	<0.001	–0.194	0.012	–15.758	0.824	0.804, 0.844	<0.001
Radiation													
	Yes	0.081	0.010	7.726	1.084	1.062, 1.107	<0.001	0.072	0.010	7.037	1.075	1.054, 1.097	<0.001
Chemotherapy													
	Yes	1.126	0.010	117.563	3.083	3.026, 3.141	<0.001	1.028	0.010	107.262	2.796	2.744, 2.849	<0.001
DX.bone													
	Yes	–0.288	0.011	–27.262	0.750	0.735, 0.766	<0.001	–0.328	0.010	–34.230	0.721	0.707, 0.734	<0.001
DX.brain													
	Yes	–0.149	0.012	–12.903	0.862	0.843, 0.881	<0.001	–0.248	0.011	–22.156	0.780	0.763, 0.797	<0.001
DX.liver													
	Yes	–0.445	0.013	–33.009	0.641	0.624, 0.658	<0.001	–0.366	0.012	–30.575	0.693	0.677, 0.710	<0.001
DX.lung													
	Yes	–0.003	0.011	–0.284	0.997	0.975, 1.019	0.777	—	—	—	—	—	—
MaritalStatus													
	Others	–0.221	0.010	–21.203	0.802	0.786, 0.818	<0.001	–0.121	0.009	–12.982	0.886	0.870, 0.902	<0.001
Income													
	USD 50,000–USD 75,000	0.156	0.015	10.238	1.168	1.134, 1.204	<0.001	0.051	0.015	3.334	1.053	1.021, 1.085	<0.001
	USD 75,000+	0.382	0.016	23.911	1.465	1.420, 1.511	<0.001	0.135	0.017	7.911	1.145	1.107, 1.184	<0.001
County													
	Metropolitan	0.192	0.014	13.435	1.212	1.179, 1.247	<0.001	0.063	0.015	4.203	1.065	1.034, 1.097	<0.001

Abbreviations: OS, overall survival; AFT, accelerated failure time; Std.Err., Standard Error; TR, time ratio; CI, confidence interval; LUAD, lung adenocarcinoma; LUSC, lung squamous cell carcinoma; PSM, propensity score matching; Hist, histology; DX, diagnosis of metastasis; Post, post-immunotherapy period.

**Table 3.** **Accelerated failure time (AFT) model for cancer-specific survival (CSS) in patients with metastatic NSCLC after PSM**.

Variables		Univariable						Multivariable					
		Estimate	Std.Err.	Z value	TR	95% CI	*p* value	Estimate	Std.Err.	Z value	TR	95% CI	*p* value
Age		–0.019	0.001	–35.198	0.982	0.981, 0.983	<0.001	–0.011	0.000	–22.761	0.989	0.988, 0.990	<0.001
Era													
	Post	0.257	0.011	23.706	1.293	1.266, 1.321	<0.001	0.255	0.009	27.084	1.290	1.267, 1.314	<0.001
Sex													
	Male	–0.261	0.011	–24.079	0.770	0.754, 0.787	<0.001	–0.232	0.010	–24.132	0.793	0.778, 0.808	<0.001
Race													
	Black	–0.031	0.016	–1.913	0.970	0.940, 1.001	0.056	0.010	0.014	0.721	1.010	0.982, 1.040	0.471
	Others	0.503	0.018	28.658	1.654	1.598, 1.712	<0.001	0.350	0.016	22.347	1.420	1.377, 1.464	<0.001
Laterality													
	Right	–0.027	0.011	–2.354	0.974	0.952, 0.996	0.019	–0.039	0.010	–3.933	0.962	0.943, 0.981	<0.001
	Others	–0.146	0.022	–6.621	0.864	0.827, 0.902	<0.001	–0.069	0.019	–3.611	0.933	0.898, 0.969	<0.001
Hist													
	LUSC	–0.352	0.014	–25.150	0.703	0.684, 0.723	<0.001	–0.222	0.013	–17.598	0.801	0.781, 0.821	<0.001
	Others	–0.357	0.014	–24.628	0.700	0.680, 0.720	<0.001	–0.195	0.013	–15.299	0.823	0.802, 0.843	<0.001
Radiation													
	Yes	0.060	0.011	5.569	1.062	1.040, 1.085	<0.001	0.067	0.011	6.253	1.069	1.047, 1.091	<0.001
Chemotherapy													
	Yes	1.118	0.010	112.449	3.060	3.000, 3.120	<0.001	1.025	0.010	103.022	2.786	2.732, 2.841	<0.001
DX.bone													
	Yes	–0.311	0.011	–28.461	0.732	0.717, 0.748	<0.001	–0.345	0.010	–34.766	0.708	0.695, 0.722	<0.001
DX.brain													
	Yes	–0.172	0.012	–14.441	0.842	0.822, 0.862	<0.001	–0.264	0.012	–22.770	0.768	0.751, 0.786	<0.001
DX.liver													
	Yes	–0.466	0.014	–33.460	0.627	0.610, 0.645	<0.001	–0.380	0.012	–30.644	0.684	0.668, 0.701	<0.001
DX.lung													
	Yes	–0.003	0.012	–0.259	0.997	0.974, 1.020	0.796	—	—	—	—	—	—
MaritalStatus													
	Others	–0.211	0.011	–19.548	0.810	0.793, 0.827	<0.001	–0.114	0.010	–11.780	0.892	0.875, 0.909	<0.001
Income													
	USD 50,000–USD 75,000	0.146	0.016	9.222	1.157	1.121, 1.193	<0.001	0.038	0.016	2.399	1.039	1.007, 1.072	0.016
	USD 75,000+	0.377	0.017	22.721	1.457	1.411, 1.505	<0.001	0.126	0.018	7.106	1.134	1.096, 1.175	<0.001
County													
	Metropolitan	0.195	0.015	13.163	1.216	1.181, 1.252	<0.001	0.068	0.016	4.374	1.071	1.038, 1.104	<0.001

Abbreviations: CSS, cancer-specific survival; AFT, accelerated failure time; TR, time ratio; CI, confidence interval; LUAD, lung adenocarcinoma; LUSC, lung squamous cell carcinoma; PSM, propensity score matching; Hist, histology; DX, diagnosis of metastasis; Post, post-immunotherapy period.

### 3.4 Interaction Assessments

**Fig. 2.**
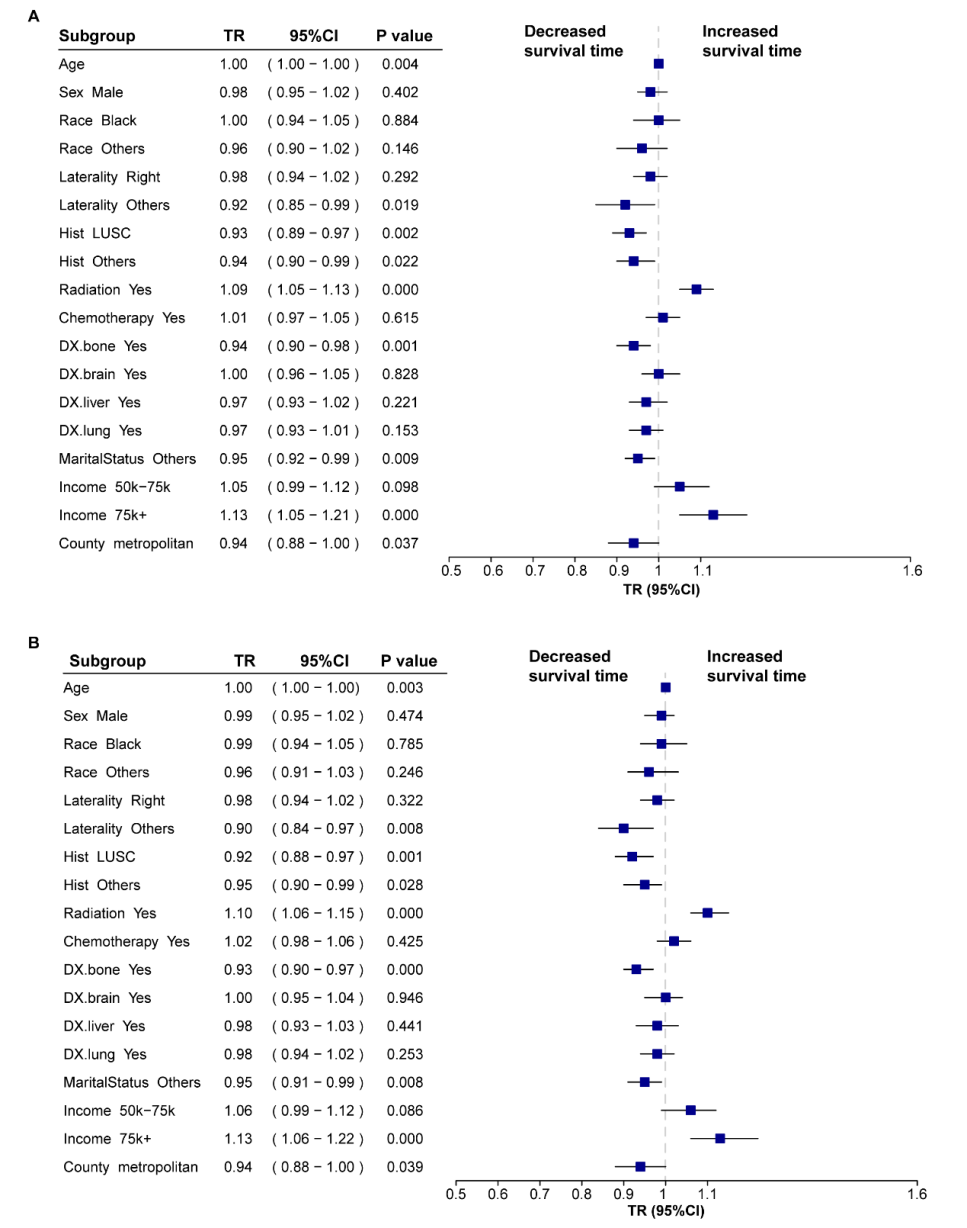
**Forest plots of subgroup interaction assessments for survival time in metastatic non-small cell lung cancer (NSCLC)**. (A) Forest plot for overall survival (OS). (B) Forest plot for cancer-specific survival (CSS). Time ratios (TR) and 95% confidence intervals (CI) were derived from the accelerated failure time (AFT) model. Each subgroup reflects the interaction term “Era (Post) × Variable” and represents the differential survival effect in the post-immunotherapy era compared to the pre-immunotherapy era. Abbreviations: Hist, histology; LUSC, lung squamous cell carcinoma; DX, diagnosis of metastasis; TR, time ratio; CI, confidence interval.

By analyzing the interaction between the period and other variables, we further evaluated the temporal evolution of variables that influence the survival outcomes of patients with metastatic NSCLC. In the post-immunotherapy periods, unspecified tumor locations, non-LUAD, bone metastases, and non-marital status were associated with shorter OS and CSS (all *p* < 0.05). Conversely, the importance of receiving radiation therapy and having a median household income above USD 75,000 was reinforced in enhancing patient OS and CSS (all *p* < 0.05) (Fig. 2).

### 3.5 CVD Mortality Risk

**Fig. 3.**
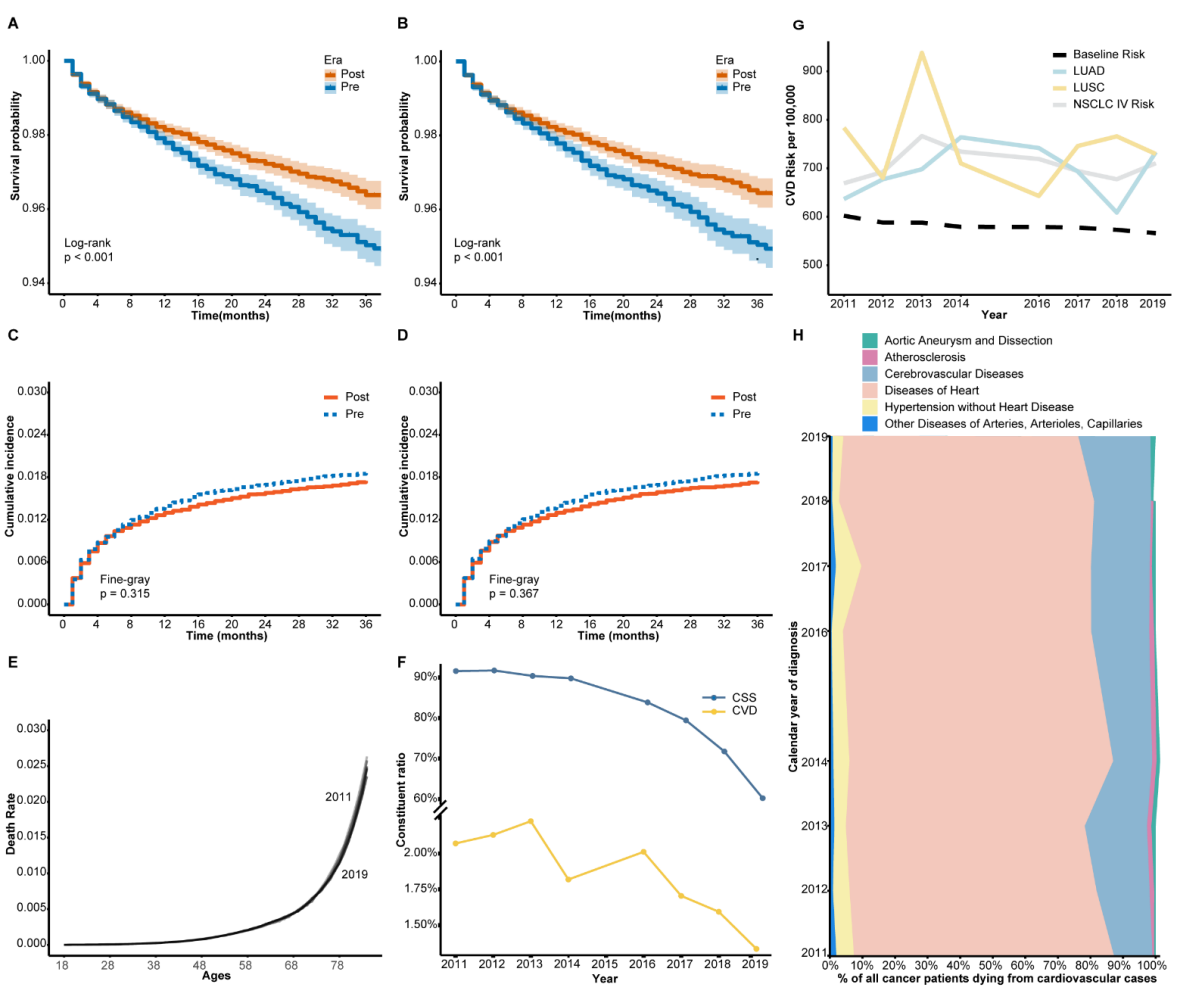
**Cardiovascular disease (CVD) mortality and cancer-specific survival (CSS) trends in patients with metastatic non-small cell lung cancer (NSCLC) during the pre- and post-immunotherapy periods**. (A) Kaplan–Meier curves of CVD-specific survival before propensity score matching (PSM). (B) Kaplan–Meier curves after PSM. (C) Cumulative incidence of CVD mortality before PSM using competing risk analysis. (D) Cumulative incidence of CVD mortality after PSM. (E) Age-specific CVD mortality rates in the general U.S. population aged 18–84, derived from the CDC-WONDER database. Data are shown for two time periods: 2011–2014 and 2016–2019, aligned with the pre- and post-immunotherapy eras in the SEER NSCLC cohort. These curves provide reference baselines for interpreting age-related CVD mortality patterns. (F) Annual trends in cancer-specific survival (CSS) and CVD mortality rates in metastatic NSCLC (SEER database). (G) Annual CVD mortality risk for different NSCLC subtypes (LUAD, LUSC, and overall stage IV NSCLC) vs. baseline population risk (CDC-WONDER). (H) Proportional distribution of CVD-related causes of death among metastatic NSCLC patients (2011–2019). Abbreviations: CSS, cancer-specific survival; CVD, cardiovascular disease; LUAD, lung adenocarcinoma; LUSC, lung squamous cell carcinoma; PSM, propensity score matching; CDC-WONDER, Centers for Disease Control and Prevention Wide-Ranging Online Data for Epidemiologic Research; SEER, Surveillance, Epidemiology, and End Results Program.

Kaplan-Meier analyses were conducted pre- and post-PSM, demonstrating higher CVD survival rates post-immunotherapy (*p* < 0.001, Fig. 3A,B). However, competitive risk analysis did not reveal significant differences in cumulative CVD mortality between the periods (*p* = 0.315, Fig. 3C; *p* = 0.367, Fig. 3D). Examination of the general U.S. population CVD data from CDC-WONDER showed an age-related increase in mortality, with an overall decreasing trend over time (Fig. 3E). In the SEER database, there was a downward trend in the incidence of both CSS and CVD mortality (Fig. 3F). When adjusting for age distribution weights, the CVD mortality risk for stage IV NSCLC patients exceeded that of the general U.S. population (Fig. 3G). The composition of the CVD subgroup did not change significantly in the pre-and post-immunotherapy periods (Fig. 3H).

## 4. Discussion

The approval of nivolumab marked a pivotal transition into the immunotherapy period for metastatic NSCLC treatment [21]. While previous studies have extensively explored the efficacy and safety of immunotherapy, this study is the first to analyze the evolution of survival determinants in metastatic NSCLC in the pre-and post-immunotherapy period and to update the CVD mortality risk assessments [13, 15, 22]. It reveals that the divergent effects of pathology type, tumor metastasis site, marital status, radiotherapy, and economic status on survival outcomes were significantly more pronounced in the post-immunotherapy period. In addition, while no period-specific differences in CVD mortality risk were identified, it remained elevated in metastatic NSCLC patients compared to the general US population. The study highlights the need to update clinical practice and health management strategies and to promote multidisciplinary integrated management in the period of immunotherapy.

Compared with previous studies [23, 24], this study observed a decline in OS for both LUSC and LUAD patients (**Supplementary Table 3**)—especially notable in the post-immunotherapy period—this outcome may be attributed to the study’s only focus on Stage IV NSCLC patients, and including those who received no treatment [3]. Nevertheless, the survival benefit of patients in the post-immunotherapy period was still significantly higher.

While earlier studies have assessed the efficacy of immunotherapy, our study did not attempt to determine its direct impact due to the absence of treatment-specific data in the SEER database. Instead, we used the term “post-immunotherapy period” as a temporal marker reflecting population-level treatment evolution, including—but not limited to—the broader adoption of immune checkpoint inhibitors. Accordingly, the observed improvements in survival should be interpreted in the context of multifactorial systemic advances, and no causal attribution to immunotherapy was made.

Multivariate AFT analyses identified vital variables that significantly impacted survival prognosis in patients with metastatic NSCLC, with consistent directionality and approximate effect strengths on OS and CSS (Tables 2,3). Radiation therapy and chemotherapy, as essential components of the first-line treatment regimen for metastatic NSCLC, provide significant survival benefits. However, extrapulmonary metastases still pose an additional survival risk to patients [25]. Notably, the dose-response relationship, influenced by lung volume differences in radiation therapy, may increase survival risks for patients with non-left lateralized [26]. In addition, consistent with most prior studies, patients with stage IV NSCLC with non-LUAD pathologic types face more significant survival challenges, partly because of the lower response rate to novel therapies, such as targeted therapies, in this group. Currently, epidermal growth factor receptor (EGFR) and kirsten rat sarcoma viral oncogene homolog (KRAS) mutations associated with targeted therapies predominantly occur in LUAD, and the study and identification of non-LUAD driver genes have lagged relatively behind [27]. Although the emergence of immunotherapy has mitigated this problem to some extent, the search for effective second-line therapeutic strategies after the failure of first-line therapy remains an urgent problem.

Our study describes the evolutionary trajectory of survival determinants in two periods, where advanced age, non-LUAD, bone metastases, and non-marital status increase the survival risk. At the same time, the protective effects of radiotherapy and higher economic levels are elevated. This indicates that although improvements in medical technology, such as radiotherapy, are extending survival for NSCLC patients, these advancements also highlight how variations in treatment regimen suitability across different patient subgroups—LUAD versus non-LUAD, with or without bone metastases—are intensifying disparities in survival outcomes. Furthermore, it’s critical to acknowledge that not all identified factors are amenable to clinical intervention or control for the benefit of metastatic NSCLC patients. Non-medical factors, including age, sex, marital status, economic standing, and geographical location, also play a pivotal role in the holistic management of these patients. The differentiation in social age structure [5], economic level [8], and emotional support [28] presented by these factors exacerbates the divergent effects of patient survival outcomes, suggesting that non-medical factors are worth considering when constructing an overall management framework for patients with metastatic NSCLC [7].

Several previous studies have evaluated the potential CVD mortality risk of multiple treatment regimens for metastatic NSCLC, including radiation, chemotherapy, immunotherapy, and combination therapies, but conclusions have varied significantly between studies [11, 12]. With future changes in the combination of treatment regimens and improvements in monotherapy techniques, treatment-related CVD mortality risk will continue to be evaluated. Our study provides another observational perspective, analyzing the evolutionary trajectory of CVD mortality risk across different periods in metastatic NSCLC through comparative analysis. This trajectory of change has been previously analyzed from this perspective by Bishnoi *et al*. [29] and Jiao *et al*. [30], who came to similar conclusions: the risk of CVD mortality in metastatic NSCLC did not increase in the post-immunotherapy period and even declined compared with the pre-immunization period. Our study provides additional evidence that CVD mortality risk was not significantly different in the pre- and post-immunotherapy periods but showed a downward trend, and it was noted that the composition of CVD subgroups remained stable. However, caution is warranted when interpreting the apparent ’reduction’ in CVD mortality risk among patients with metastatic NSCLC. On the one hand, as treatment regimens for metastatic NSCLC have become more complex and personalized, patients tend to receive longer-term cardiac medications and cardiovascular monitoring, which may reduce the incidence of malignant CVD mortality risk. On the other hand, the potential combined risk of patients was assessed before receiving chemotherapy, radiotherapy, and immunotherapy, which may have precluded patients with higher cardiovascular risk from receiving these treatments [31]. However, these explanations still need to be further validated by future studies. Given that patients with metastatic NSCLC still have a higher risk of CVD mortality compared to the general U.S. population, it is crucial to develop cardio-oncology teams to provide holistic support and continuous surveillance for cancer patients undergoing various treatment regimens.

This study has several limitations. First, it was a retrospective analysis based on the SEER and CDC-WONDER databases, which may introduce selection bias. Second, although immunotherapy has become a key component of first-line treatment for stage IV NSCLC in the post-immunotherapy era, the SEER database lacks detailed information on immunotherapy, chemotherapy, and radiation therapy. As a result, treatment-specific analyses could not be performed. Third, data on targeted therapies, comorbidities, and risk factors such as smoking and alcohol use were unavailable, limiting our ability to adjust for potential confounders. Moreover, as with treatment and comorbidity data, cause-of-death information in SEER is derived from death certificates and may be subject to misclassification. For example, patients with advanced NSCLC may be recorded as having died from lung cancer even when the proximate cause was non-oncologic. Finally, due to reporting delays, our analyses may not fully reflect trends after 2020.

## 5. Conclusions

By analyzing the evolutionary trajectory of survival factors and CVD mortality risk in patients with metastatic NSCLC in the pre-and post-immunotherapy periods, this study found that the divergent effects of factors such as pathological type, location of tumor metastasis, marital status, radiotherapy, and economic status on survival outcomes were enhanced in the post-immunotherapy period. Although there was no significant difference in CVD mortality risk between groups, a downward trend was observed. Further studies are needed to analyze the underlying mechanisms quantitatively. Considering the complexity and personalization of metastatic NSCLC treatment, narrowing the differences in survival benefits across patient subgroups and enhancing multidisciplinary collaboration in oncological cardiology may be a continuing concern for the future.

## Availability of Data and Materials

The data presented in the manuscript are derived from the SEER databases (https://seer.cancer.gov/) and the CDC-WONDER database (https://wonder.cdc.gov/).

## Supplementary Material

Supplementary material associated with this article can be found, in the online version, at https://doi.org/10.31083/RCM39296.
