# Supplementary material for: A Tale of Two Periods: The Evolution of Determinants and CVD Mortality Risk in Metastatic NSCLC
Source: Rev Cardiovasc Med. 2025 Sep 28;26(9):39296. doi: 10.31083/RCM39296 (PMC12516739; doi:10.31083/RCM39296)
Supplement: Supplementary file 1 [file 2153-8174-26-9-39296-s1.docx]

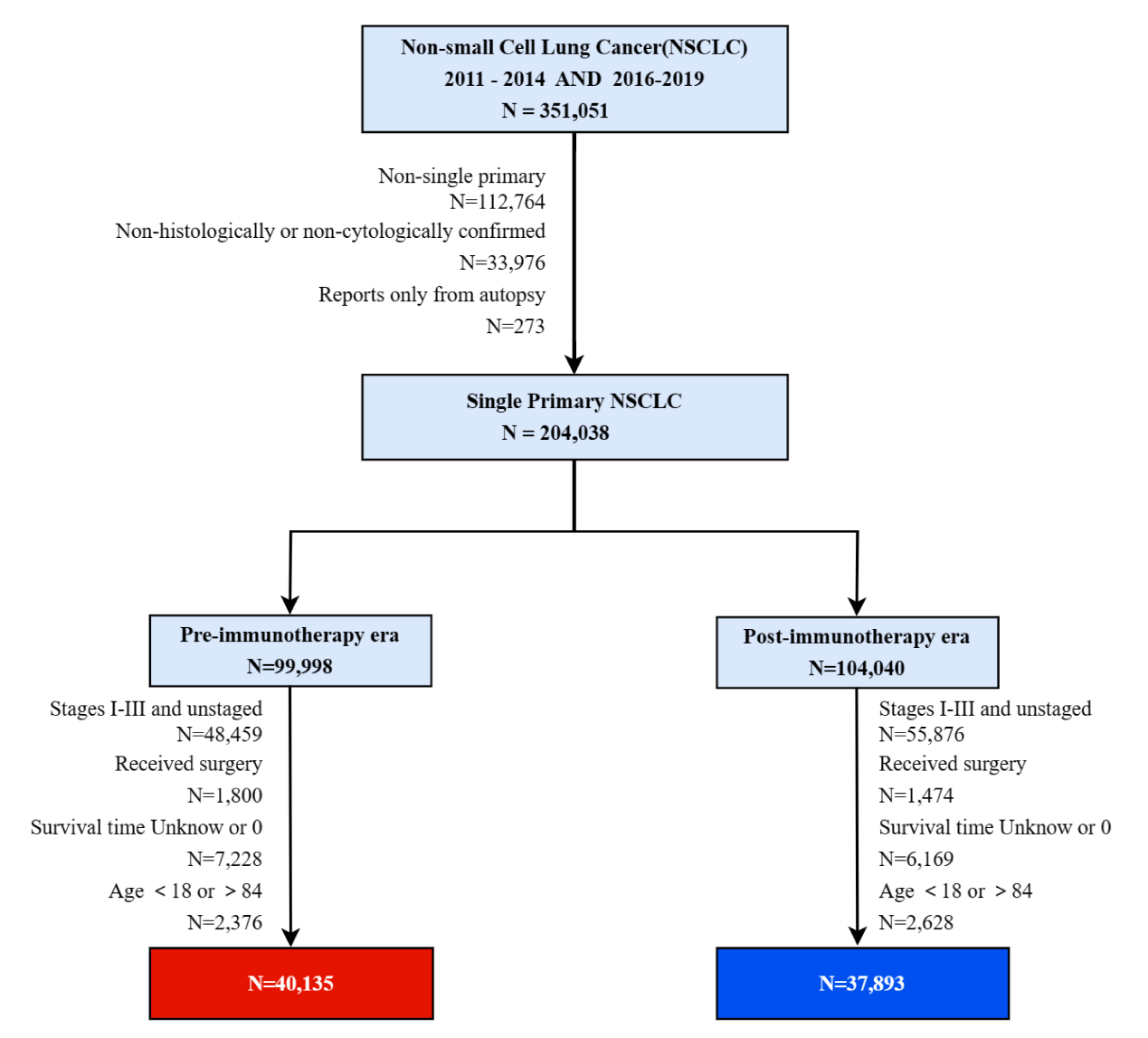


**Supplementary Fig. 1. Flowchart of inclusion and exclusion criteria**





**Supplementary Fig. 2. Q-Q Plot of Deviance Residuals for Lognormal AFT Model**


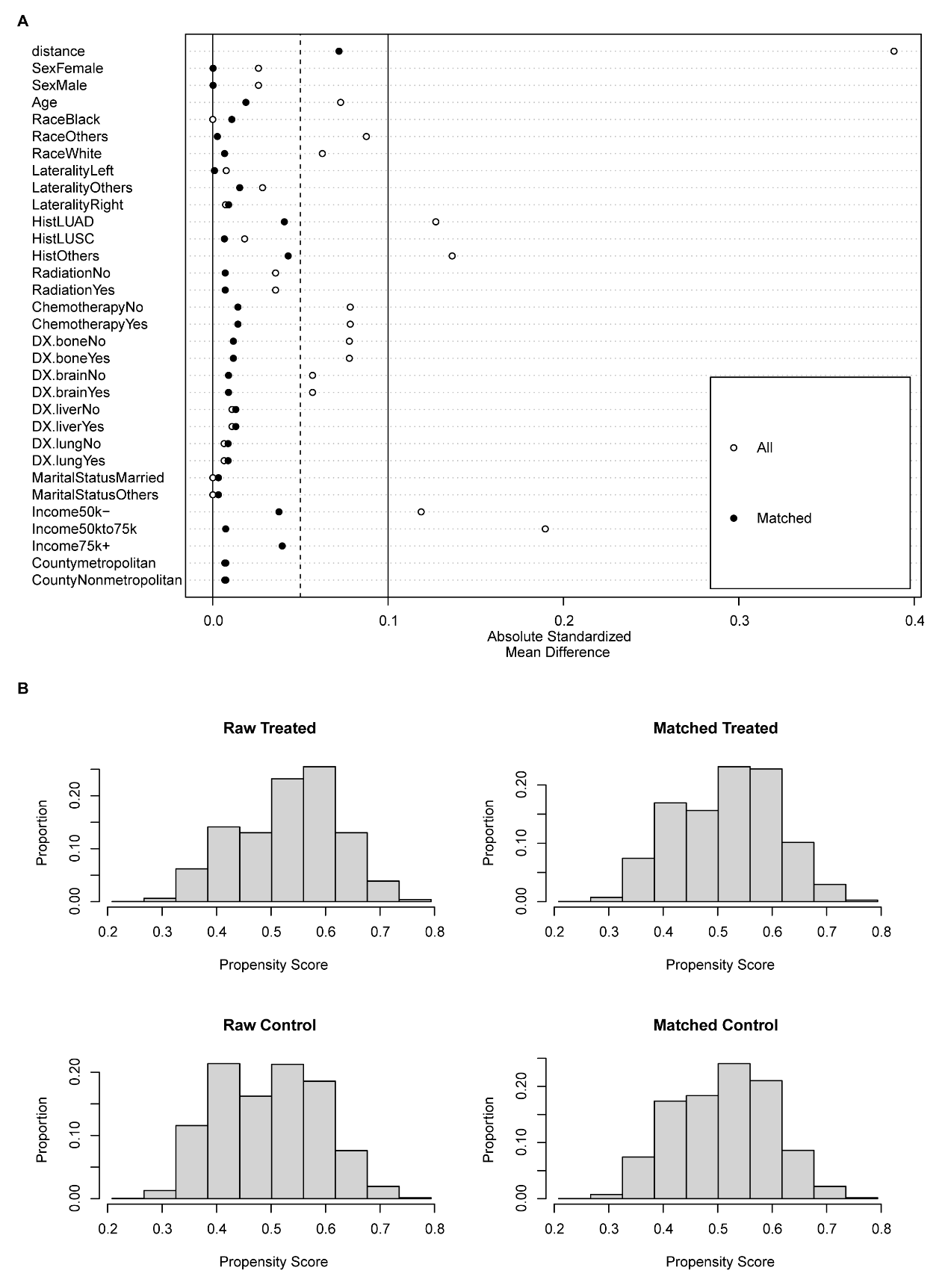


**Supplementary Fig. 3.**  **PSM balance assessment.A** Love Plot,**B** Distribution Histogram

**Supplementary Table 1** Morphology Codes of Lung Cancer Based on ICD-O-3 Classification

| Pathological Subtype | ICD-O-3 Morphology Codes |
| --- | --- |
| Small-cell lung cancer | 8002/3、8041/3，8042/3，8043/3，8044/3，8045/3 |
| Adenocarcinoma | 8050/3，8140/3，8141/3，8144/3，8201/3，8250/3-8255/3，8260/3，8290/3，8310/3，8323/3，8333/3，8480/3，8481/3，8490/3，8507/3，8550/3，8570/3，8574/3，8576/3 |
| Squamous cell carcinoma | 8052/3，8070/3-8074/3，8083/3，8084/3，8123/3 |

**Supplementary Table 2** Comparison of Akaike and Bayesian Information Criteria (AIC and BIC) for Candidate AFT Models

| Distribution Type | AIC values | BIC values |
| --- | --- | --- |
| Lognormal | 396053.2 | 396235.5 |
| Weibull | 410680.4 | 410862.7 |
| Exponential | 412704.8 | 412878.0 |

Abbreviations: The lognormal AFT model demonstrated the best fit based on the lowest AIC and BIC values among all tested distributions.

**Supplementary Table 3** Stratified Survival Outcomes by Histological Subtype (LUAD vs. LUSC) in Pre- and Post-Immunotherapy Eras

| **Variables** | **LUAD** | | |  | | | **LUSC** | | |
| --- | --- | --- | --- | --- | --- | --- | --- | --- | --- |
|  | Pre | Post | *P* value* | |  | Pre | | Post | *P* value |
| OS |  |  | <0.001 | |  |  | |  | <0.001 |
| 6-mo(%) | 50.9(50.2-51.5) | 57.2(56.5-57.8) |  | |  | 40.7(39.6-42.0) | | 46.3(45.1-47.5) |  |
| 12-mo(%) | 32.7(32.1-33.4) | 42.3(41.6-43.0) |  | |  | 21.4(20.4-22.4) | | 28.3(27.3-29.5) |  |
| 24-mo(%) | 16.9(16.4-17.4) | 27.2(26.6-27.8) |  | |  | 7.6(7.0-8.3) | | 13.6(12.7-14.5) |  |
| 36-mo(%) | 9.9(9.5-10.4) | 19.2(18.6-19.8) |  | |  | 4.1(3.7-4.6) | | 8.7(8.0-9.6) |  |
| MST(mo) | 7(7,7) | 9(9,9) |  | |  | 5(5,5) | | 6(5,6) |  |
| CSS |  |  | <0.001 | |  |  | |  | <0.001 |
| 6-mo(%) | 52.8(52.1-53.5) | 59.5(58.8-60.2) |  | |  | 43.2(42.0-44.4) | | 48.9(47.7-50.2) |  |
| 12-mo(%) | 34.7(34.1-35.4) | 44.9(44.2-45.6) |  | |  | 23.7(22.6-24.8) | | 31.1(29.9-32.3) |  |
| 24-mo(%) | 18.7(18.1-19.2) | 29.9(29.3-30.6) |  | |  | 9.1(8.4-9.9) | | 16.0(15.0-17.0) |  |
| 36-mo(%) | 11.4(11.0-11.9) | 21.8(21.2-22.5) |  | |  | 5.3(4.8-6.0) | | 10.8(9.8-11.8) |  |
| MST(mo) | 7(7,7) | 10(10,10) |  | |  | 5(5,6) | | 6(6,7) |  |

Abbreviations: OS, Overall Survival; CSS, Cancer-Specific Survival; MST, Median Survival Time; LUAD, Lung Adenocarcinoma; LUSC, Lung Squamous Cell Carcinoma; mo, months.**P* < 0.001 for Pre- vs. Post-era comparison within each histological subtype (log-rank test).Data are presented as percentage (95% confidence interval) or median (IQR).
